# Biotransformation of keratin waste to amino acids and active peptides based on cell-free catalysis

**DOI:** 10.1186/s13068-020-01700-4

**Published:** 2020-04-01

**Authors:** Zheng Peng, Xinzhe Mao, Juan Zhang, Guocheng Du, Jian Chen

**Affiliations:** 1 School of Biotechnology, Jiangnan University, 1800 Lihu Road, Wuxi, 214122 China; 2 National Engineering Laboratory for Cereal Fermentation Technology, Jiangnan University, 1800 Lihu Road, Wuxi, 214122 China; 3 Key Laboratory of Industrial Biotechnology, Ministry of Education, Jiangnan University, 1800 Lihu Road, Wuxi, 214122 China

**Keywords:** Keratin waste, Keratinase, Enzymatic hydrolysis, Amino acids and peptides

## Abstract

**Background:**

Keratin is the primary constituent of the vertebrate epidermis and epidermal appendages, as well as the main waste product generated during poultry processing from feathers, hair, scales, nails, etc. Keratin is generally hard, stubborn and difficult to hydrolyze; however, it is also inexpensive and contains more than 85% protein. Currently, tens of millions of tons of keratin waste are produced each year worldwide; however, no effective methods for the recovery of keratin waste have been reported thus far, making such research urgent. Keratinase has been reported to be useful for keratin waste recovery; however, nearly all keratinases are unable to hydrolyze keratin after they are detached from living cell systems. This may be due to low keratinase activity and lack of synergistic factors.

**Results:**

Herein, the keratinase gene from *Bacillus licheniformis* BBE11-1 was successfully expressed in *Bacillus subtilis* WB600, allowing for improved activity of the recombinant keratinase KerZ1 to 45.14 KU/mL via promoter substitution and screening of the ribosome-binding sites. Further, real-time control of temperature, pH, dissolved oxygen, and feed strategy allowed the activity of KerZ1 to reach 426.60 KU/mL in a 15-L fermenter, accounting for a 3552-fold increase compared to the wild-type keratinase (120.1 U/mL). Most importantly, we proposed a method based on the synergistic action of keratinase KerZ1 and sodium sulfite, to hydrolyze feathers into amino acids. In specific, 100 g/L of feather waste can be successfully converted into 56.6% amino acids within 12 h, while supporting the production of dozens of bioactive peptides.

**Conclusions:**

The activity of recombinant keratinase can be greatly enhanced via transcription and translational regulation in *Bacillus subtilis*. The synergistic action of keratinase and sulfite can rapidly degrade feather waste and produce amino acids and polypeptides.

## Background

With the consumption of meat products, tens of millions of tons of keratin waste are produced each year worldwide, of which feather waste accounts for approximately 8.5 million tons [1, 2]. Keratin is highly prevalent in animal coverings, including feathers, hair, scales, and nails [3, 4]. Further, the crude protein content of keratin waste is more than 85%, which is a very attractive protein reserve; however, recycling of this product poses problems that are similar to those in case of cellulose [5]. Keratin materials, divided into α-keratin and β-keratin, contain a large number of disulfide bonds, hydrogen bonds, and hydrophobic interactions, which are difficult to decompose [6–8]. High temperature, microwaving, and chemical methods (strong acid or alkali) have been explored for the decomposition and recovery of feathers. However, the violent reaction process leads to a significant loss of essential amino acids in the product, as well as enormous energy consumption, which places a notable burden on the environment [9–11].

Hence, transformation of agro-industrial wastes into valuable products has emerged as a “green” concept in sustainable industry. The discovery of keratinase revealed the potential to reuse keratin materials through biotechnological methods [12]. Keratinase is a proteolytic enzyme that specifically degrades hard keratin and is widespread in fungi and bacteria, which can break down keratin materials, converting them into soluble proteins, peptides, and amino acids [13, 14]. Keratinase has a broad spectrum of substrates, indicating its advantage for applications in degrading keratin polymers [15]. In fact, KERQ7 has shown higher levels of hydrolysis and catalytic efficiency than Basozym^®^ CS10, Korobon^®^ SC5K, and Pyrase^®^ 250MP, which currently account for the primary commercial proteases employed for bating [16]. Moreover, *Bacillus subtilis* NRC3 was reported to show higher keratinase activity under extreme conditions, such as high salinity and high temperature, completely degrading feathers within 24 h [17], while the recombinant *Bacillus amyloliquefaciens* showed enhanced feather degradation properties and shortened reaction time within 12 h [18]. However, these keratinases exhibit minimal effects after they become detached from the host bacteria, posing a major obstacle to the commercialization of keratinase.

Recently, enzyme-based resource sustainability and environmental protection processes have rapidly evolved along novel, cleaner routes in an effort to become more efficient, easy to use, and functionally focused [19, 20]. In addition, the rapid development of enzyme immobilization and protein engineering has furthered the customization of new recyclable industrial biocatalysts [21]. Keratinase, specifically, has demonstrated great potential in catalyzing keratin hydrolysis, and is, therefore, considered to be an ideal biocatalyst for the conversion of keratin waste [15, 22, 23]. However, the current use of keratinase to hydrolyze feathers is plagued by low enzyme activity and reduced processing capacity; thus, the true practical application of keratinase is yet to be recognized [24, 25]. Different research groups have sought to improve the functionality of keratinase. For instance, Yang et al. recombinantly expressed keratinase KerK in *Bacillus amyloliquefaciens* K11. Following a 60-h fermentation process, the resulting extracellular activity reached 1500 U/mL [18]. Further, Su et al. employed a recombinant *B. subtilis* keratinase mutant M7 to obtain 3040 U/mL of extracellular keratinase by continuous fermentation for 32 h in a 15-L bioreactor [26]. To this end, we also constructed a recombinant *Bacillus subtilis* strain that can efficiently express keratinase and found that we were able to rapidly process large amounts of feathers with the highly active recombinant keratinase KerZ1, to obtain a considerable amount of free amino acids and active peptides. The process of hydrolyzing feathers with ultra-high activity keratinase is quick and simple to operate, and the product yield is much higher than that in case of acid or alkali treatment. Most importantly, we have truly realized the potential for recycling of keratin waste in this simple, rapid, and green approach, which is conducive to industrial scale-up production.

## Results

### Optimize keratinase expression levels through promoter substitution

**Fig. 1:**
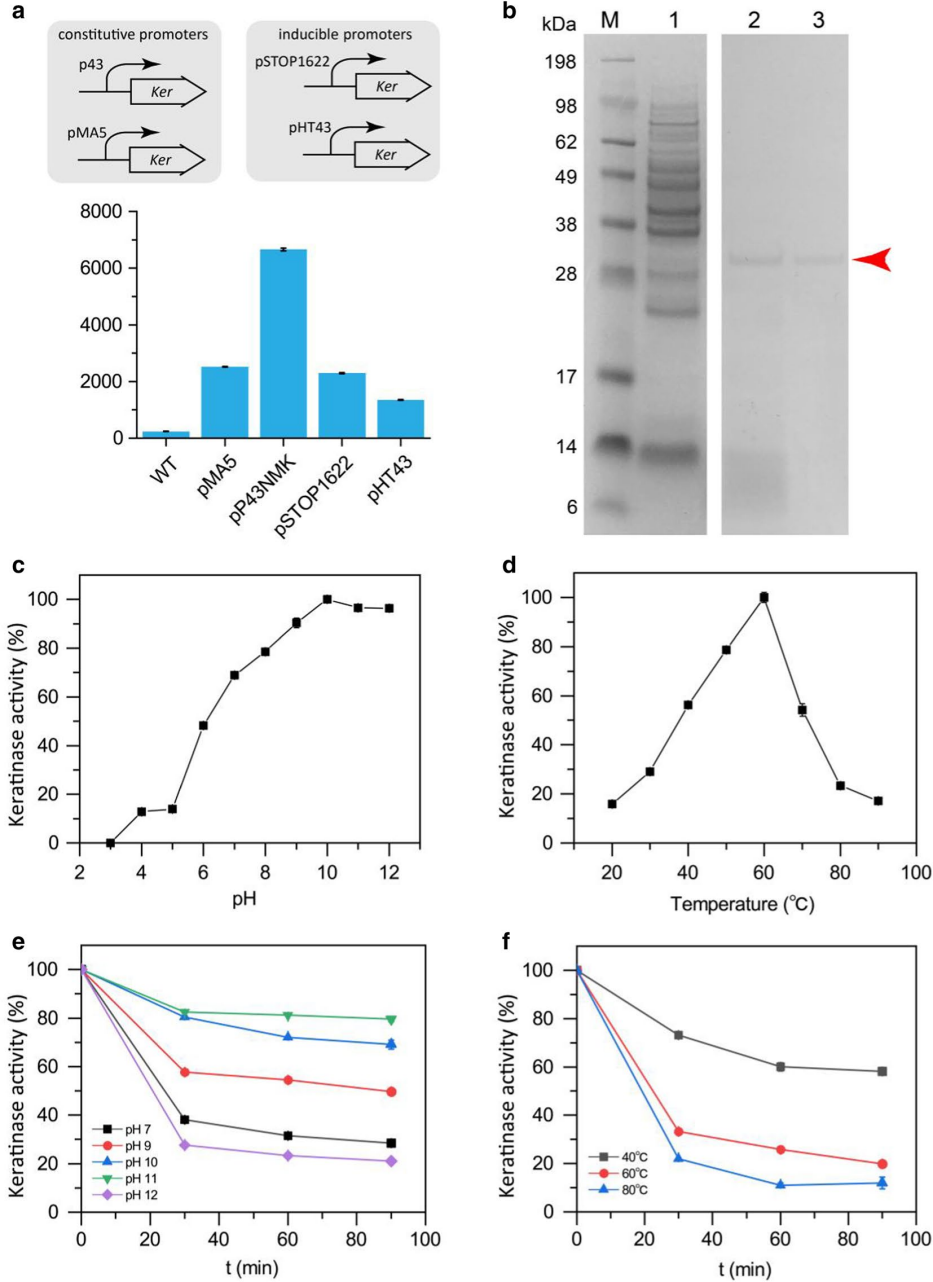
Effect of promoters on keratinase activity and the enzymatic properties of recombinant keratinase KerZ1. **a** Schematic representation of different promoters and their corresponding keratinase activities. WT: wild-type keratinase activity secreted by *Bacillus licheniformis* BBE11-1. **b** SDS-PAGE analysis of recombinant keratinase. Lane 1: The fermentation supernatant of *Bacillus subtilis* WB600, Lane 2: Recombinant keratinase KerZ1, Lane 3: Purified recombinant keratinase KerZ1. **c** Optimum pH of KerZ1. **d** Optimum temperature of KerZ1. **e** pH stability. KerZ1 was appropriately diluted with buffers of different pH and incubated at 37 °C for 90 min, after which the residual enzyme activity of keratinase was measured at an interval of 30 min. **f** Temperature stability. KerZ1 was incubated at different temperatures for 90 min. The residual enzyme activity of keratinase was then measured at an interval of 30 min

In previous studies, analysis of keratin degradation by wild-type strains showed that the activity of keratinase is directly related to the degree of keratin degradation [12, 27]. However, the activity of keratinase secreted by wild-type strains is limited. To increase the activity of keratinase, we constructed a heterologous expression system for the keratinase gene (NCBI database accession number: JX504681) in *Bacillus subtilis* with clear genomic information. Four recombinant strains were constructed based on constitutive and inducible promoters. The recombinant strains, *B. subtilis* WB600-pMA5-*ker* and *B. subtilis* WB600-pP43NMK-*ker,* were fermented for 48 h at 37 °C and 220 rpm in the initial medium. Figure 1a shows that significant differences were apparent in the expression of keratinase between the two constitutive promoters. The activity of extracellular keratinase expressed by *Bacillus subtilis* WB600-pMA5-*ker* was 2514.4 U/mL, while that of *Bacillus subtilis* WB600-pP43NMK-*ker* reached 6659.0 U/mL. Although the promoters P_HpaII_ and P_43_ carried by the pMA5 and pP43NMK plasmids, respectively, were both strong promoters and the copy number of the plasmids was high, P43 had a higher ability to initiate transcription than P_HpaII_, thus leading to a significant difference in protein expression [28, 29].

Successful expression of keratinase by the recombinants *B. subtilis* WB600-pSTOP1622-*ker* and *B. subtilis* WB600-pHT43-*ker* was based on the induction of xylose and IPTG [30]. When the cell concentration of the fermentation system reached 0.8 (OD_600_ = 0.8), xylose with a final concentration of 0.5% and IPTG with a final concentration of 0.4 mM were added, followed by incubation at 37 °C and shaking conditions of 220 rpm for 48 h. The corresponding recombinant strains had an extracellular keratinase activity of 2296.4 U/mL and 1353.6 U/mL, respectively (Additional file 1: Figure S1). Although the pHT43 plasmid contains the Theta replicon, the copy number of the plasmid is low (≤ 10), and the recombinant protein is not readily expressed in large quantities [31]. Hence, *B. subtilis* WB600-pP43NMK-*ker*, which expresses keratinase activity prominently, was chosen for further research on keratinase.

### Purification and characterization of keratinase

To facilitate the purification of recombinant keratinase KerZ1, a 6-His tag was added to the N-terminus of the mature enzyme and a recombinant strain was subsequently constructed. After 48 h of fermentation under the same conditions, the supernatant of the fermentation broth was examined by SDS-PAGE. It should be noted that with the exception of KerZ1, only a few non-target bands are concentrated in the low molecular weight region in the supernatant. However, in the supernatant of the *B. subtilis* WB600 fermentation broth containing no recombinant plasmid, there were numerous proteins with various molecular weights. These results indicate that keratinase has a broad spectrum of substrates; when KerZ1 is overexpressed, other proteins are decomposed by KerZ1 to form a protein or peptide with a smaller molecular weight [32]. Moreover, these results also demonstrate that KerZ1 has a strong capacity for degrading proteins, which is why it is widely applicable in various fields of scientific research and industries.

Based on the AKTA protein purification system (General electric, USA), KerZ1 was bound to a nickel column and eluted with 5% imidazole to obtain purified KerZ1, as presented in Fig. 1b. In addition, Fig. 1c, d shows the enzymatic properties of KerZ1, which demonstrated the highest keratinase activity at pH 10 and 60 °C. KerZ1 also exhibited excellent stability at pH 9–11, especially at pH 10 and 11 (Fig. 1e). Although KerZ1 has the most optimal activity at 60 °C, it also demonstrates excellent stability at lower temperatures of 40 °C, which is advantageous for storage of KerZ1 (Fig. 1f).

### Rationally designed translation initiation sequence to improve keratinase expression

**Fig. 2:**
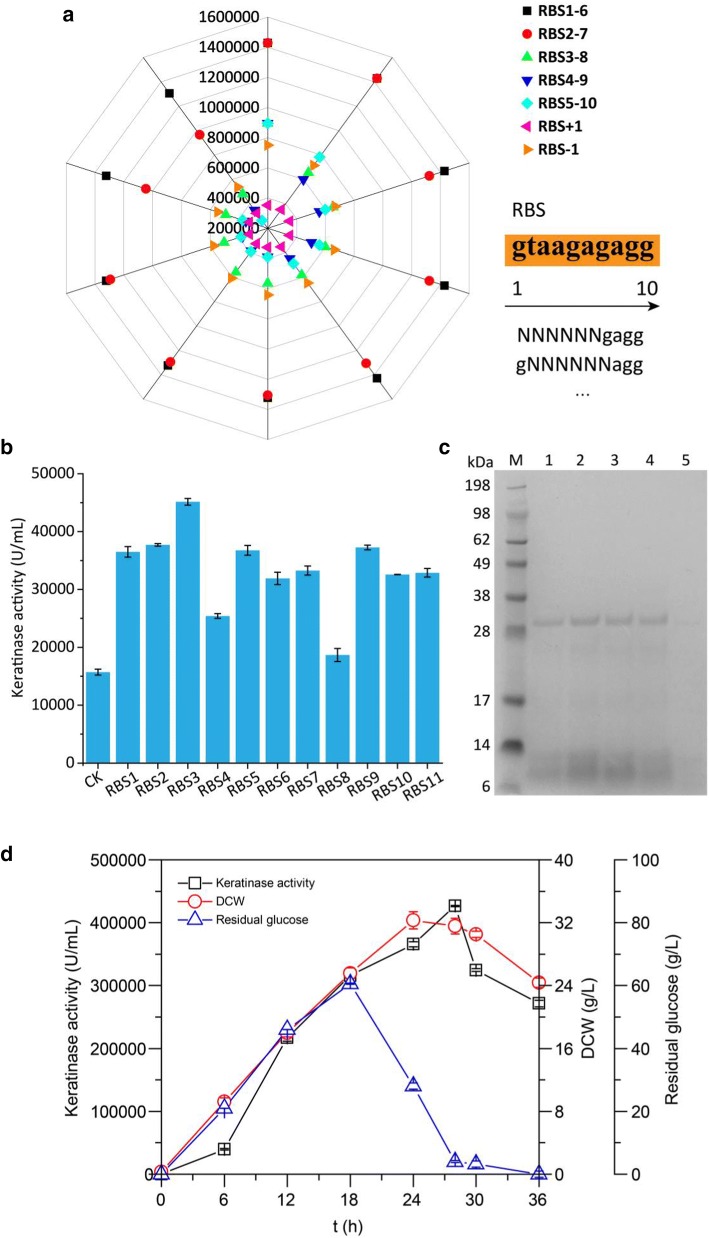
Effects of ribosome binding on translation initiation rate and keratinase activity in the corresponding mutant. **a** Prediction of the keratinase translation level using the RBS Calculator v2.0 (https://salislab.net/software/forward). **b** Keratinase activity corresponding to RBS mutants. **c** Analysis of keratinase expression by SDS-PAGE. Lane 1: RBS1, Lane 2: RBS2, Lane 3: RBS3, Lane 4: RBS9, Lane 5: Keratinase expressed by a recombinant strain containing the original RBS sequence served as a control (CK). **d** Batch fermentation of mutant RBS3 in 15 L fermenter

Sodium dodecyl sulfate–polyacrylamide gel electrophoresis (SDS-PAGE; Fig. 1b) of recombinant keratinase KerZ1 demonstrated that the extracellular expression of keratinase was limited, which is detrimental to its large-scale production and application. The efficiency of translation initiation in bacterial cells is determined by the performance of the ribosome-binding site (RBS); while translation initiation affects translation efficiency by more than 100-fold [33], appropriate promoter and RBS sequences can greatly increase the expression level of the protein [34]. To increase keratinase expression, the RBS was rationally designed to improve translation efficiency via prediction of the keratinase translation level using the RBS Calculator v2.0 (https://salislab.net/software/forward) [35]. As the RBS sequence was moved closer to the translation region, its influence on translation initiation efficiency decreased [35] (Fig. 2a). Therefore, a library containing 4096 mutants was constructed via imposing a saturation mutation (GNNNNNNAGG) upstream of the keratinase gene sequence from the − 13 to − 18 region based on the original RBS sequence 5′-GTAAGAGAGG-3′ in the p43NMK plasmid. We then selected 3 times the number of theoretical transformants to achieve 95% coverage. Eleven mutants (Table 1) with a predicted Δ*G*_total_ between − 10 and − 15 were selected for further shake flask fermentation. The keratinase activity of the 11 mutants exceeded 15,000 U/mL, and the most superior mutant, RBS3, showed a keratinase activity of 45.14 KU/mL (Fig. 2b). SDS-PAGE analysis for keratinase expression revealed that the mutants RBS1, RBS2, RBS3, and RBS9 exhibited significant improvement relative to the original recombinant enzyme (Fig. 2c). The above results indicate that our strategy for improving the expression level of KerZ1 at the translational level was successful.

**Table 1:** Sequence, translation initiation rate, and ΔG_tot_ of the RBS mutants

Name	RBS mutant sequence (5′→3′)	Translation initiation rate (a.u.)	Δ*G*_total_ (kcal/mol)
WT	GTAAGAGAGG	136,321.8	− 4.15
RBS1	GAGGGGGAGG	1,323,117.0	− 15.51
RBS2	GAATGGGAGG	261,800.7	− 11.91
RBS3	GAGAAGGAGG	1,293,676.2	− 15.45
RBS4	GAGGAGGAGG	1,305,372.8	− 15.47
RBS5	GTAAGAGAGG	136,321.8	− 10.45
RBS6	GGAGGGGAGG	965,565.5	− 14.80
RBS7	GTAAGAGAGG	136,321.8	− 10.45
RBS8	GGGGGGGAGG	1,323,117.0	− 15.51
RBS9	GGATAGGAGG	667,591.9	− 13.98
RBS10	GGGAGGGAGG	866,709.0	− 14.56
RBS11	GAAAGGTAGG	237,121.7	− 11.68

### Process optimization and scale-up in 15-L fermenter

Previous studies have successfully expressed keratinase in *B. subtilis* WB600 and have greatly increased the level of enzyme activity; however, they remain unable to meet the growing needs of industrial production and commercialization. The use of larger reactors to further increase the production of keratinase and to test the stability of the fermentation process is, therefore, an important property to measure the industrialization capacity of the enzyme. The recombinant strain RBS3 was inoculated at a percentage of 5% (v/v) in a 15-L fermenter to produce keratinase using the optimized medium (Additional file 1: Figure S2). The fermentation was carried out by maintaining pH at 7.0 via automatic addition of phosphoric acid and ammonia, keeping the dissolved oxygen (DO) above 30% by controlling DO associating with agitation speed, and feeding glucose at a constant rate of 28.8 g/L/h within 6–18 h for high-density fermentation. As a result, the DCW of the culture broth was 29.13 g/L following continuous fermentation for 28 h, and the yield and productivity of KerZ1 reached 426.60 KU/mL and 15.24 KU/mL/h, respectively (Fig. 2d). Further, the keratinase activity of KerZ1 was 3552 times higher than that of wild-type keratinase and 128.1 times higher than that of the initial recombinant keratinase, which is the highest yield reported thus far. This high yield of keratinase also indicated the suitability of *Bacillus subtilis* as a host for KerZ1 production.

### Keratinase ability to hydrolyze feathers

**Fig. 3:**
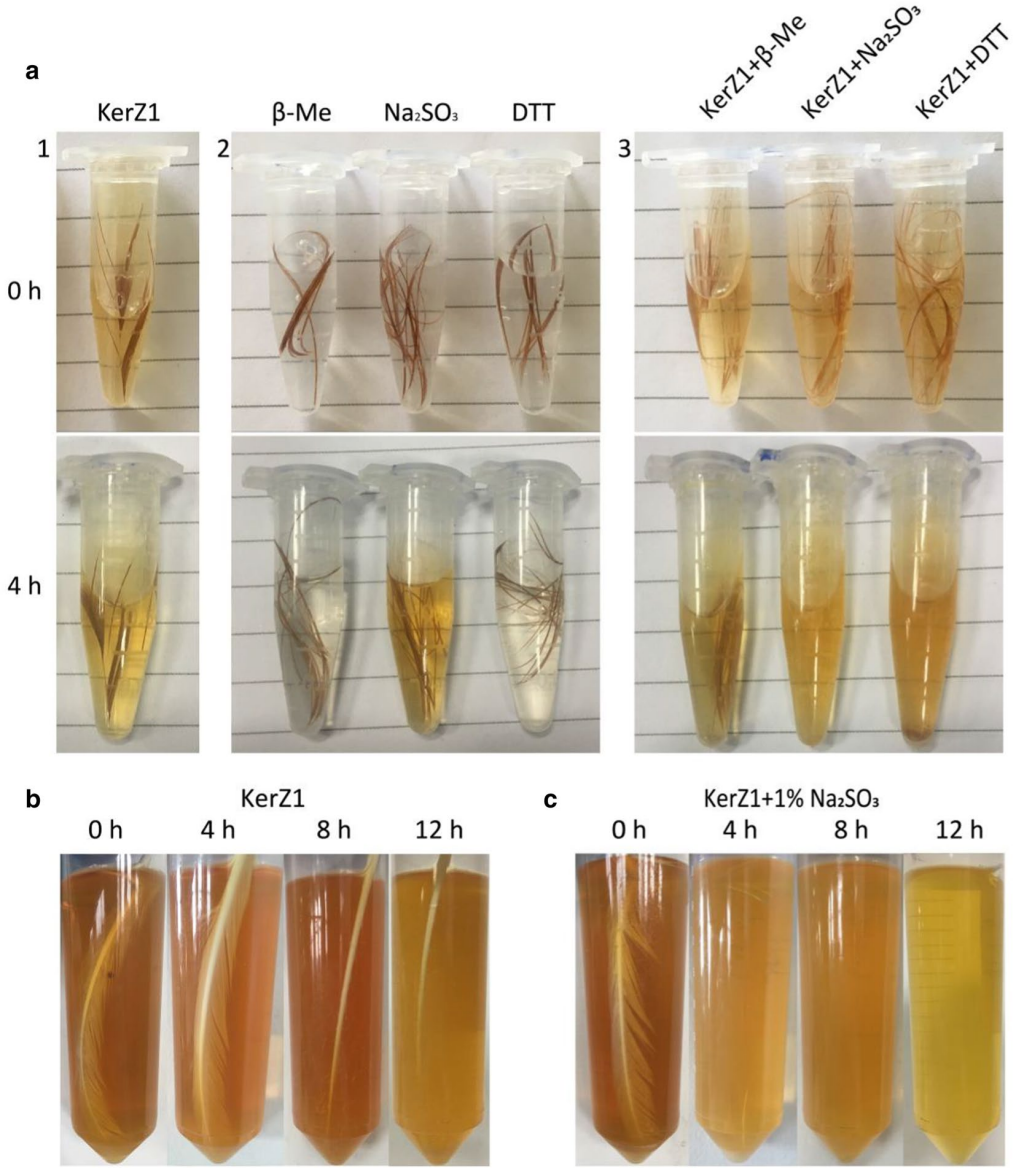
Synergetic effect of chemical reducing agent on degradation of feathers by KerZ1. **a** Effect of reducing agent on keratinase-hydrolyzing feathers. a1: Feathers were treated with KerZ1 (45.14 KU/mL) at 60 °C for 4 h; a2: feathers were treated with reducing agent β-Me, sodium sulfite and dithiothreitol at a final concentration of 0.1% at 60 °C for 4 h; a3: feathers were treated with KerZ1 containing 0.1% reducing agent β-Me (β-mercaptoethanol), sodium sulfite and dithiothreose (DTT) at 60 °C for 4 h. **b** Feathers were treated with KerZ1 (426.60 KU/mL) at 60 °C for 12 h. **c** Feathers were treated with KerZ1 (426.60 KU/mL) containing 1% sodium sulfite at 60 °C for 12 h

The process of microbial degradation of keratin involves three essential steps, namely denaturation, decomposition, and transamination [13, 36, 37]. Denaturation is critical, and involves opening the dense disulfide bond structure of keratin. This study assessed the efficiency of three reducing agents in vitro to help open the disulfide bond. β-Mercaptoethanol, sodium sulfite, and dithiothreitol (DTT) were added to the fermentation system of keratinase KerZ1 (45.14 KU/mL) to promote feather hydrolysis. Figure 3a shows that 0.1% sodium sulfite or DTT combined with KerZ1 degraded the feathers into fragments and soluble products. However, neither the individual reducing agents alone nor the recombinant keratinase with a high enzyme activity degraded the feathers. Furthermore, the most apparent change that occurred following the individual actions of these compounds was the partial fading of the feathers, suggesting that denaturation, i.e., disulfide bond cleavage, is essential for the degradation of keratin. In addition, due to the high toxicity of DTT, sodium sulfite, which is environmentally friendly and safe, was chosen to degrade the feathers in synergy with KerZ1.

Figure 3b shows that KerZ1 (426.60 KU/mL) with high enzymatic activity degraded a large proportion of the feathers during incubation at 60 °C for 12 h, leaving only the scapus. To define the optimal conditions for enzymatic hydrolysis, we first optimized the initial pH of the enzymatic hydrolysis system and the amount of sodium sulfite added. The results showed that an initial pH of 7 and addition of 1% sodium sulfite yielded the most effective result (Additional file 1: Figure S3). When KerZ1, which showed high enzyme activity, and 1% sodium sulfite were incubated with a feather for 4 h, nearly the entire feather was hydrolyzed, leaving behind only a small amount of feather fragments. The solution in the hydrolysis system became clear as the reaction progressed toward 12 h (Fig. 3c). These results are novel in that it has not yet been reported, to our knowledge, that keratinase is capable of effectively hydrolyzing feathers so thoroughly in such a short time period [38, 39]. These results reveal that a single keratinase with sufficiently high enzyme activity can hydrolyze feathers, and that the synergistic effect of sodium sulfite enhances the ability of keratinase to degrade feathers. Interestingly, the color of both systems gradually became lighter as the degradation process progressed, indicating the presence of active substances that induce fading in the hydrolysate [40].

**Fig. 4:**
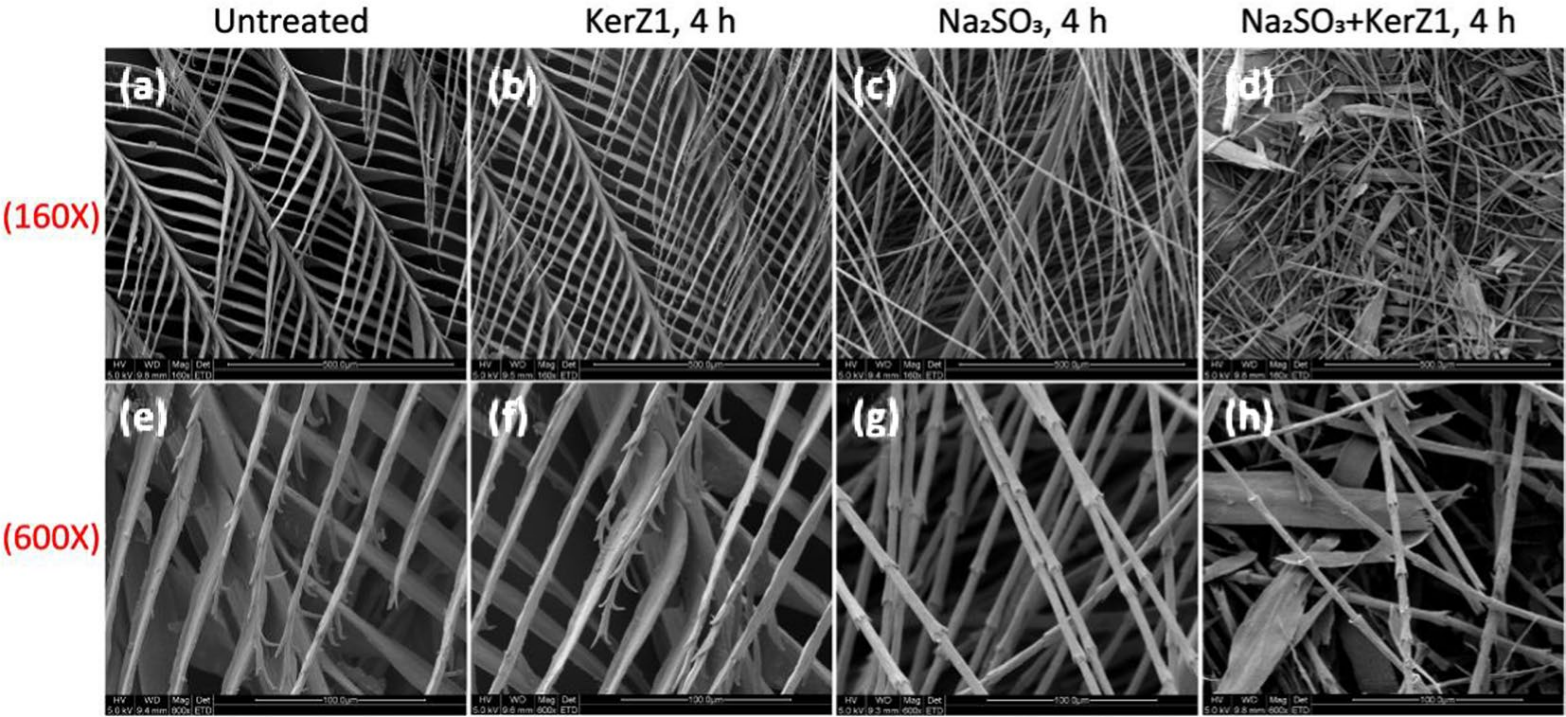
Scanning electron microscopy images of feathers. **a**, **e** Untreated feathers. **b**, **f** Feathers were treated with KerZ1 (45.14 KU/mL) at 60 °C for 4 h. **c**, **g** Feathers were treated with 0.1% sodium sulfite at 60 °C for 4 h. **d**, **h** Feathers were treated with KerZ1 (45.14 KU/mL) containing 0.1% sodium sulfite at 60 °C for 4 h

Figure 4 demonstrates the change of state in the feathers after 4 h under different treatments as evaluated by scanning electron microscopy. When treated with 0.1% sodium sulfite alone, the plumes became fine but did not break. After treatment with KerZ1 and sodium sulfite, the feathers became deformed and fractured. In addition, treatment with low-activity KerZ1 alone did not significantly alter the structure of the feathers.

### Feather hydrolysate analysis

Analysis of the types of amino acids as well as the content of the feather hydrolysate (0.12 g) generated via synergism of KerZ1 and 1% sodium sulfite is shown in Table 2. Complete hydrolysis of the feathers resulted in amino acid production with a conversion rate of 49.3%. Among them, the contents of glutamic acid, alanine, tyrosine, phenylalanine, leucine, and lysine were relatively high.

**Table 2:** Amino acid content in feather hydrolysate

Amino acid	Concentration (mg/L)	
	Supernatant	Hydrolysate
asp	27.93 ± 1.57	130.80 ± 3.94
glu	122.86 ± 0.55	380.78 ± 15.42
ser	7.72 ± 0.89	40.28 ± 1.56
his	6.38 ± 1.21	76.58 ± 3.67
gly	8.91 ± 0.12	94.30 ± 6.19
thr	25.70 ± 2.44	136.36 ± 5.28
arg	23.44 ± 0.18	19.80 ± 0.31
ala	138.23 ± 4.36	247.44 ± 5.59
tyr	55.57 ± 1.38	301.76 ± 14.27
cys-s	23.34 ± 0.28	28.58 ± 0.59
val	531.75 ± 10.55	337.62 ± 16.20
met	204.99 ± 2.77	247.16 ± 4.63
phe	401.76 ± 8.54	577.54 ± 17.48
ile	45.25 ± 0.45	105.62 ± 7.19
leu	50.30 ± 2.11	220.06 ± 8.32
lys	154.67 ± 5.17	360.48 ± 14.29
Total	1828.79	3305.16

Supernatant means the fermentation supernatant; hydrolysate means the products from feather hydrolysis

**Fig. 5:**
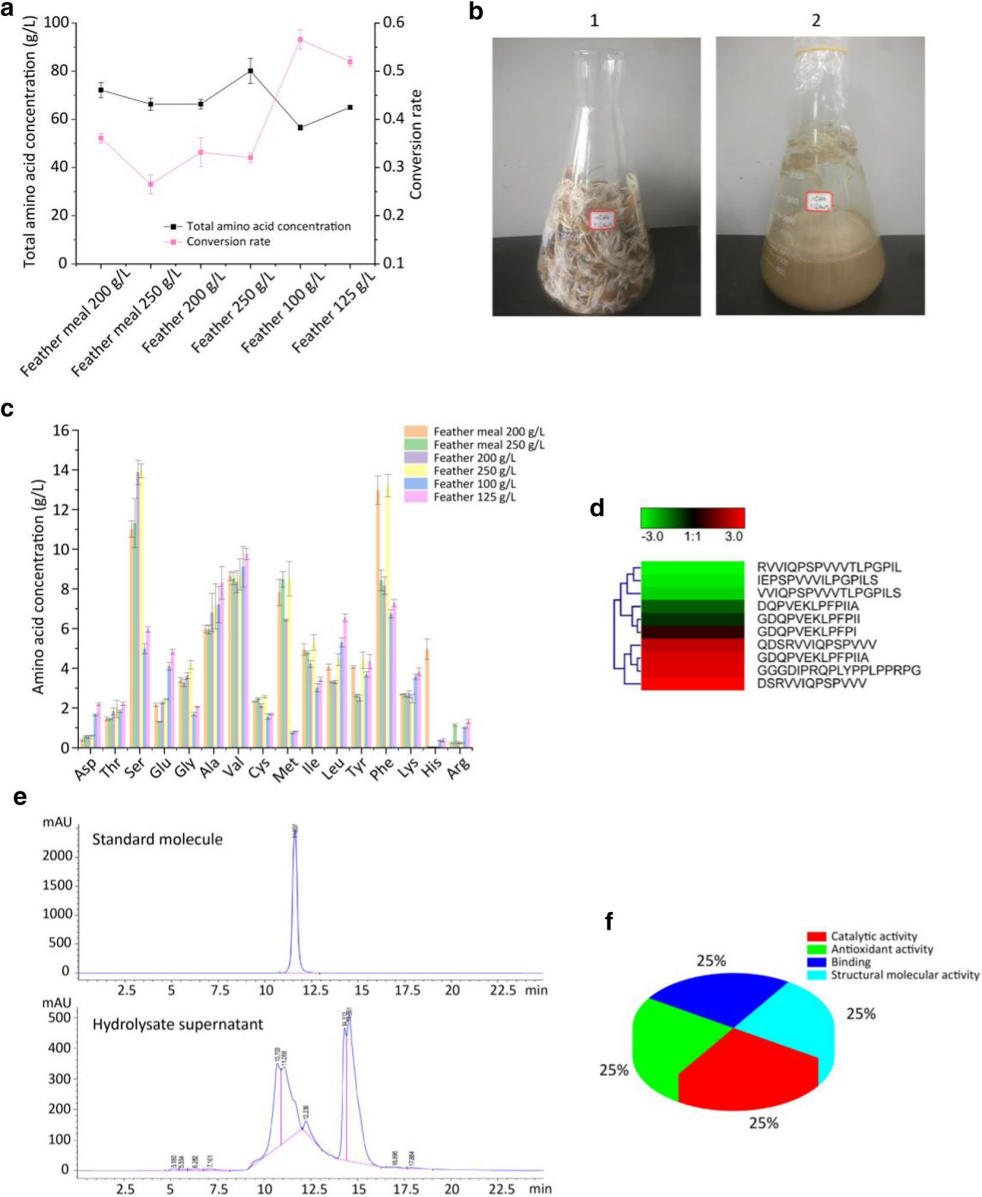
Optimization of KerZ1-hydrolyzed feather system and analysis of hydrolysates. **a** Optimization of feather addition and feather morphology. **b** Feather shape display. 1: 100 g untreated feather, 2: 100 g feather treated with keratinase. **c** Contents of 16 common amino acids in hydrolysates obtained from different hydrolysis systems. **d** Types of peptides identified from hydrolysates obtained from a hydrolysis system containing 100 g/L of feathers. **e** Molecular weight distribution of peptides in the hydrolysate obtained from a hydrolysis system containing 100 g/L of feathers. **f** Possible biological functions corresponding to the peptides obtained from the database comparison

To maximize the conversion rate of the product, we optimized the amount and shape (feather or feather meal) of feathers, and the results show that when the amount of feathers was 100 g/L, the conversion rate of amino acids reached a maximum of 56.6% (Fig. 5a, b). Moreover, the wide variety of amino acids demonstrates the enormous potential of hydrolysates as feed protein additives (Fig. 5c). The hydrolysate also contains a mixed short peptide with a molecular weight of approximately 1.3 kDa (Fig. 5e), and mass spectrometry results revealed up to 12 active peptides that exhibited catalytic activity and antioxidant activity (Fig. 5d, f).

## Discussion

The severe shortage of protein resources has motivated researchers to seek cheap and sustainable proteins, and to produce new functional materials in an environmentally friendly way. Keratin is a very valuable yet difficult-to-recycle fibrous protein that is a by-product of various meat- and poultry-processing operations [1, 41]. Currently, identifying feed additives to replace expensive fishmeal requires acidic or alkaline hydrolysis to dissolve insoluble keratin and release amino acids [10, 42]. However, these methods do not address the issues surrounding the process complexity, energy investment, poor product absorption, and use of organic reagents [10, 43]. Hence, in the current study, we optimized the promoter, RBS, and fermentation conditions to obtain the most active keratinase to date via expression in *Bacillus subtilis*; this keratinase is capable of completely hydrolyzing feathers in a short period of time. In addition, the fermentation time required to achieve these results was shorter than those reported in previous studies, which is conducive to the industrialization of this enzyme.

In this study, although the optimum catalytic temperature of keratinase KerZ1 was determined to be 60 °C, its stability at this temperature was suboptimal, which led to the slow progress of keratin hydrolysis in the later stages. We attempted to use a lower temperature for enzymatic hydrolysis; however, this led to reduced hydrolysis efficiency and amino acid yield. Therefore, structural analysis based on the rational design of keratinase to enhance its thermal stability is necessary. Of note, similar effects can be achieved by increasing its catalytic efficiency and shortening the catalytic cycle as much as possible.

We also found that the activity of keratinase was positively correlated with the state of cell growth during fermentation and increased linearly within 6–24 h (Fig. 2d). This may be related to the characteristics of a constitutive promoter, with a superior cell growth state resulting in higher yields. Therefore, continued optimization of fermentation parameters and feeding strategies will have the opportunity to significantly reduce cell division time, thereby reducing the entire time required for a fermentation cycle.

Although this study achieved rapid degradation of feathers via keratinase, the catalytic mechanism employed by keratinase has not yet been characterized, accounting for one of the primary issues faced by keratinase researchers [44]. Similarly, an abundance of polypeptides is present in the hydrolysate; however, the specific functions of these peptides are unknown. However, the custom production of functional peptides cannot be achieved by keratinase hydrolysis of feathers. Therefore, research on the substrate specificity and catalytic mechanism of keratinase will be of top priority for future studies.

## Conclusions

This study efficiently expressed KerZ1 in *B. subtilis* WB600 by applying rational design at the transcriptional, translational, and fermentation levels allowing for the enzymatic activity of KerZ1 to reach 426.60 KU/mL. We also propose a method for degrading feathers (100 g/L) to produce amino acids based on the synergistic effect of high-activity KerZ1 and sodium sulfite, with the conversion rate reaching 56.6%. In addition, this method is easy to use, quick, and does not generate toxic byproducts; it is also conducive to industrial scale-up production. In future studies, we plan to focus on the active peptides in feather hydrolysate, which will serve as a new, inexpensive source of functional peptides.

## Methods

### Chemicals, strains, and plasmids

All plasmid and promoter information has been added to Additional file 1: Table S1. *Bacillus licheniformis* BBE11-1 with keratinase secretion ability was screened by our group [45]. *E. coli* JM109 carrying the keratinase gene was constructed to amplify the recombinant plasmids (pMA5-*ker*, pP43NMK-*ker*, pSTOZ1622-*ker*, pHT43-*ker*), and the recombinant plasmids were then transformed into *Bacillus subtilis* WB600; thus, the strains required for this study were successfully constructed. Amplification of gene fragments and linearized vectors using the PrimeSTAR Max Premix (from TaKaRa Bio Inc., Dalian, China) was followed by recombination of genes and vectors using the ClonExpress II One Step Cloning Kit (from Vazyme Biotech., Nanjing, China). All RBS sequences were optimized using the recombinant plasmid pP43NMK-*ker* as a template, and the linearized vector was religated using the Blunting Kination Ligation (BKL) Kit (from TaKaRa Bio Inc., Dalian, China).

### Medium and culture method

LB medium (pH 7.0) was used to activate seeds and contained 10 g/L peptone, 5 g/L yeast extract, and 10 g/L NaCl. The initial medium for fermentation had an initial pH of 7.2 and contained 10 g/L tryptone, 10 g/L glucose, 3 g/L KH_2_PO_4_, 2 g/L Na_2_HPO_4_, and 0.6 g/L MgSO_4_. Optimized fermentation medium (pH 7.0) contained 10 g/L yeast extract, 20 g/L tryptone, 20 g/L sucrose, 3 g/L KH_2_PO_4_, 6 g/L Na_2_HPO_4_, and 0.3 g/L MgSO_4_. The culture conditions of the shake flask were 37 °C and 220 rpm. The feathers used in this study were untreated ordinary feathers obtained from a poultry market. The enzymatic hydrolysis process involving the feathers was simple, only requiring the mixing of the fermentation supernatant, sodium sulfite, and untreated feathers, followed by incubation at 60 °C to allow the reaction to proceed for 12 h.

### General procedure of keratinase activity assay

Keratinase activity was determined by the Folin–Ciocalteu method, as described previously with slight modifications [46]. The reaction system, containing 150 μL of 50 mM Gly/NaOH buffer (pH 9.0), 100 μL of 2.5% soluble keratin, and 50 μL of a suitably diluted enzyme solution, was incubated at 50 °C for 20 min. The reaction was terminated by adding 200 μL of 4% trichloroacetic acid (TCA) and centrifugation at 8000 rpm at room temperature for 3 min. Then, 200 μL of the supernatant was mixed with 1 mL of 4% Na_2_CO_3_ and 200 μL of Folin–Ciocalteu reagent at 50 °C for 10 min. The absorbance at 660 nm was measured and the corresponding enzyme activity was obtained by tyrosine standard curve conversion. All experiments were repeated three times and the samples from the control group were mixed with trichloroacetic acid before adding enzyme solution. The remaining operations were the same as those in case of the samples in the experimental group. In this study, one unit of keratinase activity was defined as an absorbance value increase of 0.001 units per minute at 660 nm.

### General procedure of batch fermentation in a 15-L fermenter

All lab-scale amplification experiments were performed in a 15-L automatic fermenter. The primary seed liquid was obtained by culturing glycerol bacteria or single colonies at 37 °C and 220 rpm for 15 h. These samples were transferred to 400 mL of LB and the secondary seed solution was prepared by continuous culture for 4 h under the same conditions. The secondary seed liquid was inoculated at 5% into a 15-L fermenter system containing 8.0 L of fermentation medium, and the fermentation process were conducted at 37 °C, 500 rpm agitation, and 2.0 vvm air flow rate. Dissolved oxygen was controlled at approximately 30% during fermentation by adjusting the stirring speed and air flow rate.

### Analysis of amino acids and peptides

Amino acids in the feather hydrolysate supernatants were detected with an amino acid analyzer (Hitachi L-8900, Tokyo, Japan). The sample was precipitated with 10% sulfosalicylic acid at a ratio of 1:1 for more than 4 h, centrifuged at 12,000*g* for 10 min, and the supernatant was retained. The final sample was diluted to 0.1 mM with 0.02 M hydrochloric acid. Conversion rate of the amino acids was calculated as follows:

$${\text{Amino}}\;{\text{acid conversion}}\;{\text{rate }}\left( \% \right)\, = \,100\, \times \,A/B,$$

where *B* is the dry weight of the feathers before decomposition and *A* the total amino acid content in the hydrolysate.

Peptides were detected by high-performance liquid chromatography (Agilent 1260 series, Santa Clara, CA, USA) using a gel column (TSK gel G2000SWXL 7.8 × 300 mm) for separation of the peptides by molecular weight. The mobile phase was phosphate buffer, and the flow rate was 0.8 mL/min. The detector, wavelength, and column temperature were VWD, 214 nm, and 25 °C, respectively. The type and function analysis of the peptide was performed by Wuhan Jinkairui Bioengineering Co., Ltd (Wuhan, China).

## Supplementary information

**Additional file 1.** Additional table and figures.

## Data Availability

All data generated or analyzed during this study are included in this published article and its additional files.
