# Supplementary material for: Biotransformation of keratin waste to amino acids and active peptides based on cell-free catalysis
Source: Biotechnol Biofuels. 2020 Apr 1;13:61. doi: 10.1186/s13068-020-01700-4 (PMC7110813; doi:10.1186/s13068-020-01700-4)
Supplement: Supplementary file 1 — Additional file 1. Additional table and figures. [file 13068_2020_1700_MOESM1_ESM.docx]

**Biotransformation of keratin waste to amino acids and active peptides based on cell-free catalysis**

Zheng Peng^1,3^, Xinzhe Mao^1,3^, Juan Zhang^1,3*^, Guocheng Du^1,3^, Jian Chen^1,2*^

^1^School of Biotechnology, Jiangnan University, 1800 Lihu Road, Wuxi 214122, China. ^2^National Engineering Laboratory for Cereal Fermentation Technology, Jiangnan University, 1800 Lihu Road, Wuxi 214122, China. ^3^Key Laboratory of Industrial Biotechnology, Ministry of Education, Jiangnan University, 1800 Lihu Road, Wuxi 214122, China.

*Corresponding authors Address: School of Biotechnology, Jiangnan University, 1800 Lihu Road, Wuxi 214122, China. Tel.: +86 510 85918307; fax: +86 510 85918309. E-mail addresses: jchen@jiangnan.edu.cn (J. Chen), zhangj@jiangnan.edu.cn (J. Zhang)

**Additional file**

Table S1. Strains and plasmids used in this study.

| **Names** | **Characteristics** | **Reference** |
| --- | --- | --- |
| **Strains** |  | |
| *E*. *coli* JM109 | *F’ traD36 proA*^+^*B*^+^ *laclq Δ(lacZ)M15/Δ(lac-proAB) glnV44 e14-gyrA96 relA1endA1 thi hsdR17* | Lab stock |
| *B*. *subtilis* WB600 | Missing *npr*E *apr*E *epr bpr mpr* *npr*B | Lab stock |
| **Plasmids** |  |  |
| pMA5 | Kan^r^；*B*.*subtilis* expression vector ; P_HpaII_ | Lab stock |
| pHT43 | Kan^r^；*B*.*subtilis* expression vector ; P_grac_ | Lab stock |
| pP43NMK | Kan^r^；*B*.*subtilis* expression vector ; P_43_ | Lab stock |
| pSTOP1622 | Tat^r^；*B*.*megaterium* expression vector ; P_xyl_ | Lab stock |

Figures

^
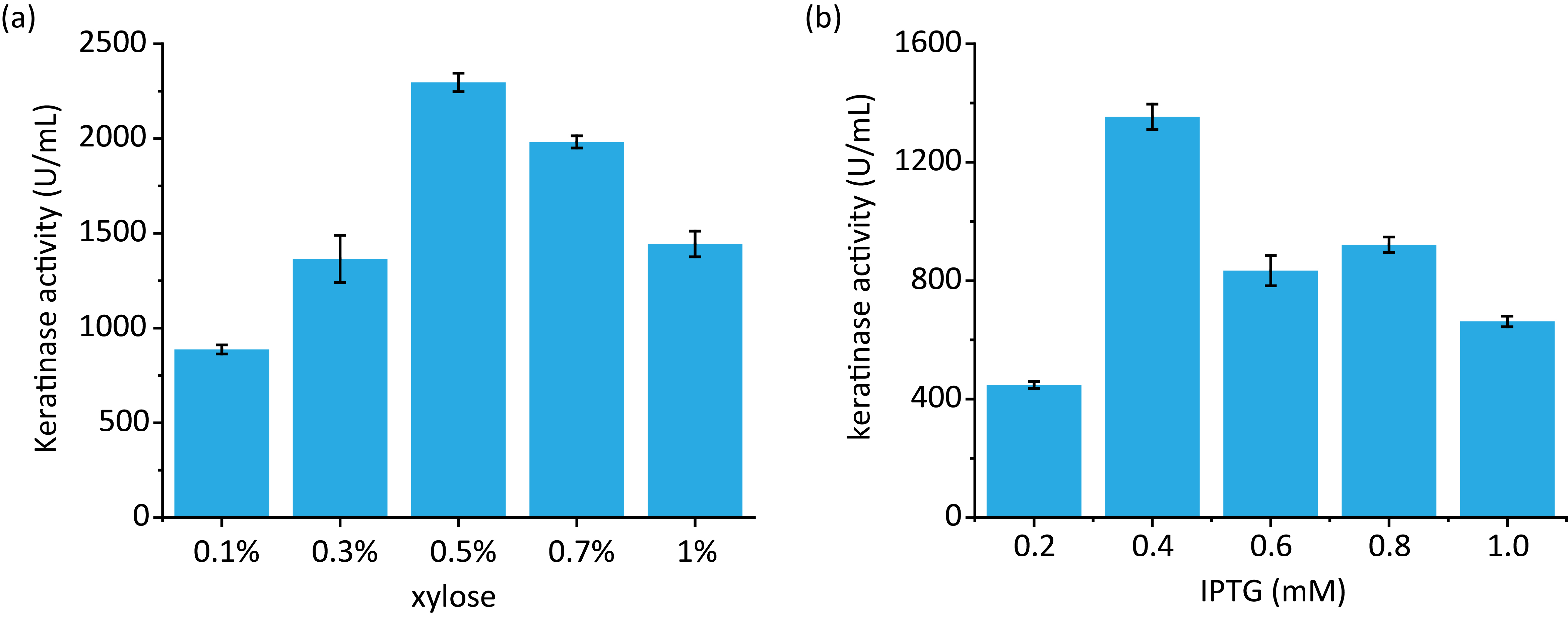
^

Figure S1 Optimization of inducer dose. (a) Optimization of final xylose concentration. (b) Optimization of final IPTG concentration.


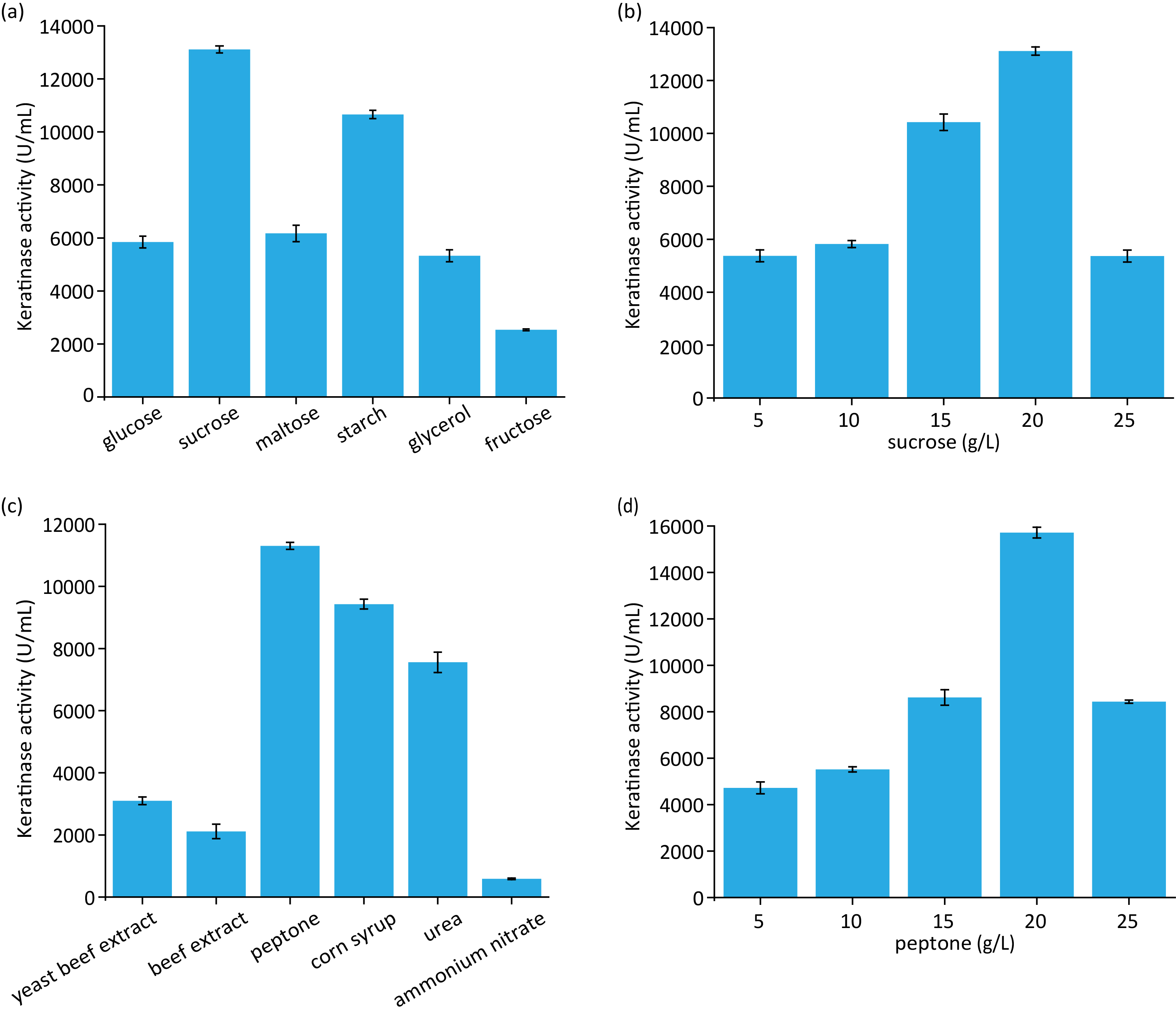


Figure S2 Optimization of carbon and nitrogen sources for fermentation media. (a) Six different carbon sources (20 g/L) (glucose, sucrose, maltose, glycerol, soluble starch and fructose). (b) Five different concentrations (5, 10, 15, 20, 25) g/L of sucrose. (c) Six different nitrogen sources (20 g/L) (beef extract, ammonium nitrate, urea, yeast extract, peptone, corn steep liquor). (d) Five different concentrations (5, 10, 15, 20, 25) g/L of peptone.


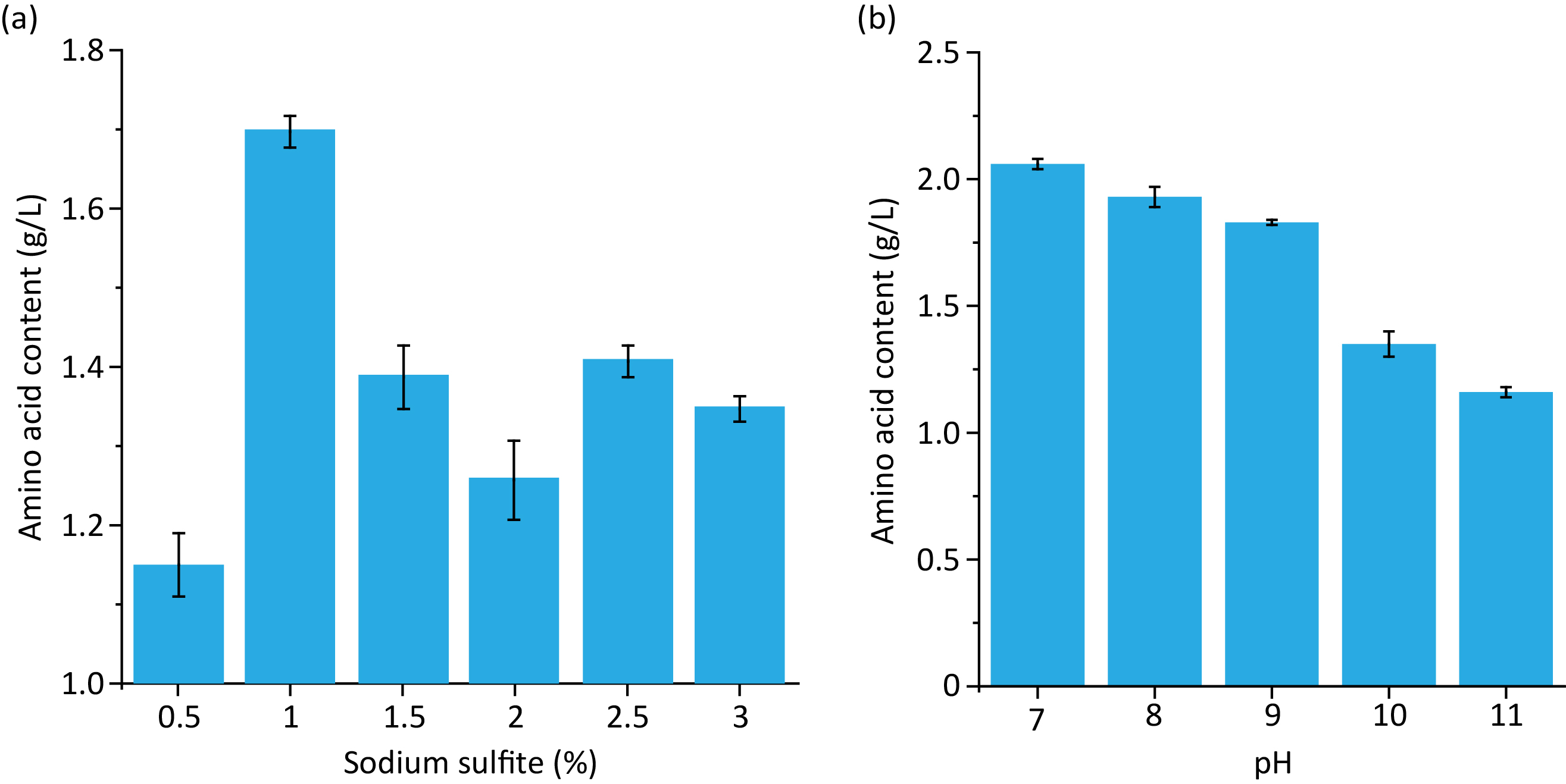


Figure S3 Optimization of enzymatic conditions. (a) Six sulfite addition concentrations (0.5%, 1%, 1.5% 2%, 2.5%, 3%). (b) Five initial pH (7, 8, 9, 10, 11).
